# Effect of Host Pupae Cryogenic, Anesthesia, Parasitoid‐to‐Host Ratios to *Scleroderma sichuanensis* the Percentage Parasitism and Numbers of Output

**DOI:** 10.1002/ece3.70649

**Published:** 2024-11-28

**Authors:** Jiangwei Zheng, Ke Li, Wei Li, Wensheng Xia, Yao Zhao, Tao Liu, Yu Peng

**Affiliations:** ^1^ Hubei Key Laboratory of Regional Development and Environmental Response, Faculty of Resources and Environmental Science Hubei University Wuhan China; ^2^ Wuhan Institute of Landscape Architecture Wuhan China; ^3^ State Key Laboratory of Biocatalysis and Enzyme Engineering, School of Life Sciences Hubei University Wuhan China

**Keywords:** anesthesia method, biological control, insect pest, parasitoid‐to‐host ratios

## Abstract

Biological control is one of the effective means of controlling forestry burrowing pests in a low‐cost and efficient way. *Scleroderma sichuanensis*, an ectoparasitic wasp, exhibits significant potential in managing burrowing pests. Currently, the mealworm *Tenebrio molitor* pupae are mainly used to many insects large‐scale indoor breeding of diet. Therefore, this study focused on the effects of cryogenically treated the mealworm *T. molitor* pupae for different times and anesthesia treatment on the percentage parasitism and the number of emergence of *S. sichuanensis* , optimum parasitism and emergence were achieved in the wasp with a parasitoid‐to‐host ratio of 2:1, anesthesia treatment of *T. molitor* pupae with ethyl ether fumigation for 1.5 h, and cold treatment of them at a temperature of −10°C. As a result, various methods of treating *T. molitor* pupae indoors were explored, and optimal conditions for indoor rearing were explored for the expansion of *S. sichuanensis* .

## Graphical abstract

At present, the pupae of *Tenebrio molitor* are mainly used to breeding *S. sichuanensis* in large numbers indoors. In this study, we summarize the previous research methods, investigated the effects of feeding conditions, parasitoid‐to‐host ratio, different anesthesia methods used on the pupae and different temperatures and duration times on the wasp emergence rate and total wasp output, we concluded the most suitable conditions for breeding of *S. sichuanensis* .

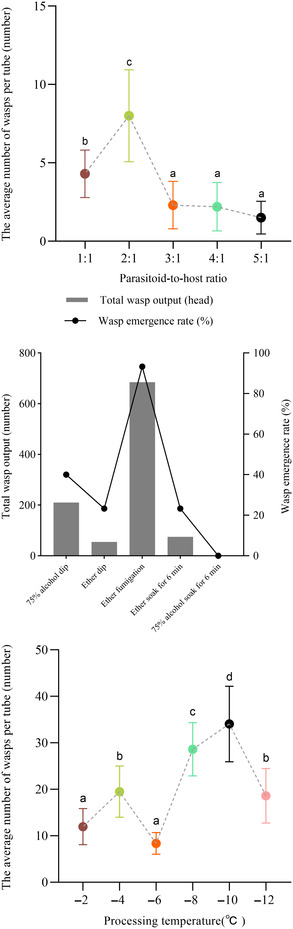

## 1 Introduction

*Scleroderma sichuanensis* (Hymenoptera, Bethylidae) is an ectoparasitic wasp that the larvae and pupae of various stem‐boring pests (Lanes and Azevedo 2008; Lupi et al. 2017; Skvarla 2018), demonstrating significant effectiveness against burrowing species such as longicorn (Golec et al. 2020; Tang et al. 2012; Yang, Wang, and Zhang 2014). It was ectoparasitic where longicorn larvae first reported 1994 in Sichuan province, China. Offering an extensive reach into the deep layers of tree trunks, *S. sichuanensis* proves invaluable in areas where chemical pesticides fall short. The wasp's excellent searchability, broad host range, high parasitism rate, robust resistance, longevity, and prolific egg‐laying capabilities, coupled with the ease of artificial reproduction, all of which are of great value for exploitation (Branco et al. 2022; Gao et al. 2016; Yang et al. 2012). At present, an established technical system for the indoor breeding of *S. sichuanensis* use of intermediate hosts and is extensively utilized for managing burrowing pests in economic forests, landscaping species, old and valuable trees, and protection forests (Ferracini et al. 2018; Men et al. 2019; Wang and Aparicio 2020; Shi et al. 2020). To optimize the propagation of *S. sichuanensis* , reliance on manual collection of longicorn larvae from felled, infested wood presents logistical challenges, with aspen proving particularly difficult to breed artificially. Thus, developing a more effective method for the wasp's mass cultivation is imperative for industrial usage in managing stem‐boring pests. Researchers have considered a variety of use alternative hosts across Lepidoptera, Coleoptera and Hymenoptera for wasp reproduction (Chardonnet et al. 2018; Ichiki et al. 2009; Li et al. 2010; Roversi et al. 2013; Wei et al. 2013).

Currently, the main alternative hosts used for large‐scale wasp breeding are mealworm *T. molitor* pupae, being used to replace the larvae of longicorn *Saperda populnea* or other Hymenoptera, Lepidoptera and Hymenoptera collected from artificial forests, as intermediate hosts for *S. sichuanensis* breeding (Zhuo et al. 2016; Wei et al. 2022). *T. molitor* pupae are highly active at room temperature, have a short pupal period, and are resistant to the sting of bethylid wasps, while the pupae are susceptible to temperature and anesthetic corrosion. Extensive research has shown that cryogenic treatment and anesthesia for it increase the wasp's the percentage parasitism and numbers of output. Therefore, screening for the optimal storage temperature and anesthesia method for *T. molitor* pupae is a demand prompt solution problem that needs to be solved during the mass expansion of bethylid wasps.

The study offers some important insights into investigated the effects of the rearing conditions for *T. molitor* , the ratio of parasitic wasp to *T. molitor* (parasitoid‐to‐host ratios), different anesthesia methods for *T. molitor* pupae, and different temperature and treatment times on the emergence rate and total output of wasp, finally, it provided technical support for the expansion of *S. sichuanensis* using substitute hosts *T. molitor* .

## 2 Materials and Methods

### 2.1 Experimental Insects

*Scleroderma sichuanensis* was collected from Hunan Xinglin Pest Control Co. Ltd. (113°01′ E, 28°14′ N), and *T. molitor* was purchased from the Wuhan Flower and Fish Market (114°30′ E, 30°61′ N). Then, these were reared individually in the finger‐shaped glass tubes (20 mm diameter, 50 mm high) in the laboratory. The larvae of *T. molitor* were fed with wheat bran for three generations. Both *S. sichuanensis* and *T. molitor* were reared in artificial climatic cabinets (CC350TLHC type; Changzhou Okefenokee instrument) that were set at 26°C ± 1°C and 60% ± 10% relative humidity under a 16:8‐h (L: D) photoperiod (Amiresmaeili et al. 2018).

### 2.2 Experiment Design

Three experimental studies have determined the percentage parasitism and numbers of total output of *S. sichuanensis* on *T. molitor* pupae under various conditions, including duration of cryogenic treatment, anesthesia, and the ratio of hosts to parasitoid wasps.

### 2.3 Experiment 1: *T. molitor* Pupae Was Fed a Mixed Diet Under Laboratory Conditions

The experiments were conducted with freshly pupated, milky white pupae of *T. molitor* . All of *T. molitor* pupae were being raised in plastic box (15 cm × 10 cm × 8 cm) in a climatic chamber under laboratory conditions (26.4°C; 43% RH, 16:8 L:D). It was been breeding wheat bran + corn flour + green feed, clean environment and good ventilation as experiment host materials. One pupa was placed in a test tube (length × diameter = 10 cm × 1 cm) and inoculated with *S. sichuanensis* . One *T. molitor* pupa was, respectively, attached to 1, 2, 3, 4, and 5 *S. sichuanensis* in parasitoid‐to‐host ratios and each treatment was repeated 30 times. The experiments were conducted in an incubator at 26°C ± 1°C (Tian and Xu 2003) and 60% ± 10% humidity to compare the wasp emergence rate and total wasp output under different wasp ratios (Abdi et al. 2021; Wei et al. 2017).

### 2.4 Experiment 2: *T. molitor* Pupae Were Different Ways Anesthesia Under Laboratory Conditions

Anesthesia methods for *T. molitor* pupae were dip, fumigation and soak. The anesthetics used were 75% alcohol and ether. The treatments were 75% alcohol dip, ether dip, alcohol soak, ether soak, and ether fumigation (Favaro et al. 2017). For the different treatments, each pupa treated with anesthesia was inducted into *S. sichuanensis* at most suitable parasitoid‐to‐host ratio, and each treatment was replicated 30 times. The wasp emergence rate and wasp output of each treatment were recorded. For the dip method, the newly pupated *T. molitor* pupae were dipped in a Petri dish containing alcohol (75%) or ether for 1 s and then removed. The pupae were placed on filter paper to dry, placed in a plastic bag and sealed for 0.5 h before receiving *S. sichuanensis* . For the soak method, the white pupae of just pupated *T. molitor* were placed into a Petri dish with alcohol (75%) and ether for 6 min, then put into a plastic bag and sealed for 0.5 h before receiving *S. sichuanensis* . For the fumigation method, 10 mL of ether was placed into a closed plastic bottle, *T. molitor* pupae was added, and then the bottle was smoked for 1.5 h before *S. sichuanensis* was added.

### 2.5 Experiment 3: *T. molitor* Pupae Was Duration of Cryogenic Treatment

A two‐factor, no‐replication test was designed considering two factors: treatment temperature (0°C, −4°C and −8°C) and duration (1, 2 and 3 days). *T. molitor* pupae treated by different temperature and duration were inoculated with *S. sichuanensis* at most suitable parasitoid‐to‐host ratio and 30 tubes were inoculated in each treatment to record the wasp emergence rate and total wasp output. The bivariate ANOVA results showed that the treatment temperature had a significant effect on the wasp output; thus, we designed a two‐factor analysis of variance (ANOVA) with different treatment temperatures (−2°C, −4°C, −6°C, −8°C, −10°C, and −12°C) for 2 days. After *T. molitor* pupae treated abovementioned condition being inoculated with *S. sichuanensis* at same parasitoid‐to‐host ratio, the wasp emergence rate and total wasp output were recorded for each treatment.

### 2.6 Data Analysis

$$\text{Wasp emergence rate}=\frac{\text{Number of offspring wasp tubes}}{\text{Number of inoculated tubes}}\times 100\%$$

The total wasp output was the sum of the wasp output of all wasp‐catching tubes. Data are expressed as the mean ± standard error. All data were analyzed by one‐way ANOVA was used for the pupation time, pupal weight, emergence rate, and wasp output of *T. monitor* pupae at different parasitoid‐to‐host ratios. Multiple comparisons of the means were performed using Tukey's HSD multiple comparisons of means, and all analyses were performed in SPSS 26.0 (SPSS Inc., Chicago, IL).

## 3 Results

### 3.1 Part 1: Experiment 1: The Most Suitable Parasitoid‐to‐Host Ratio

**FIGURE 1:**
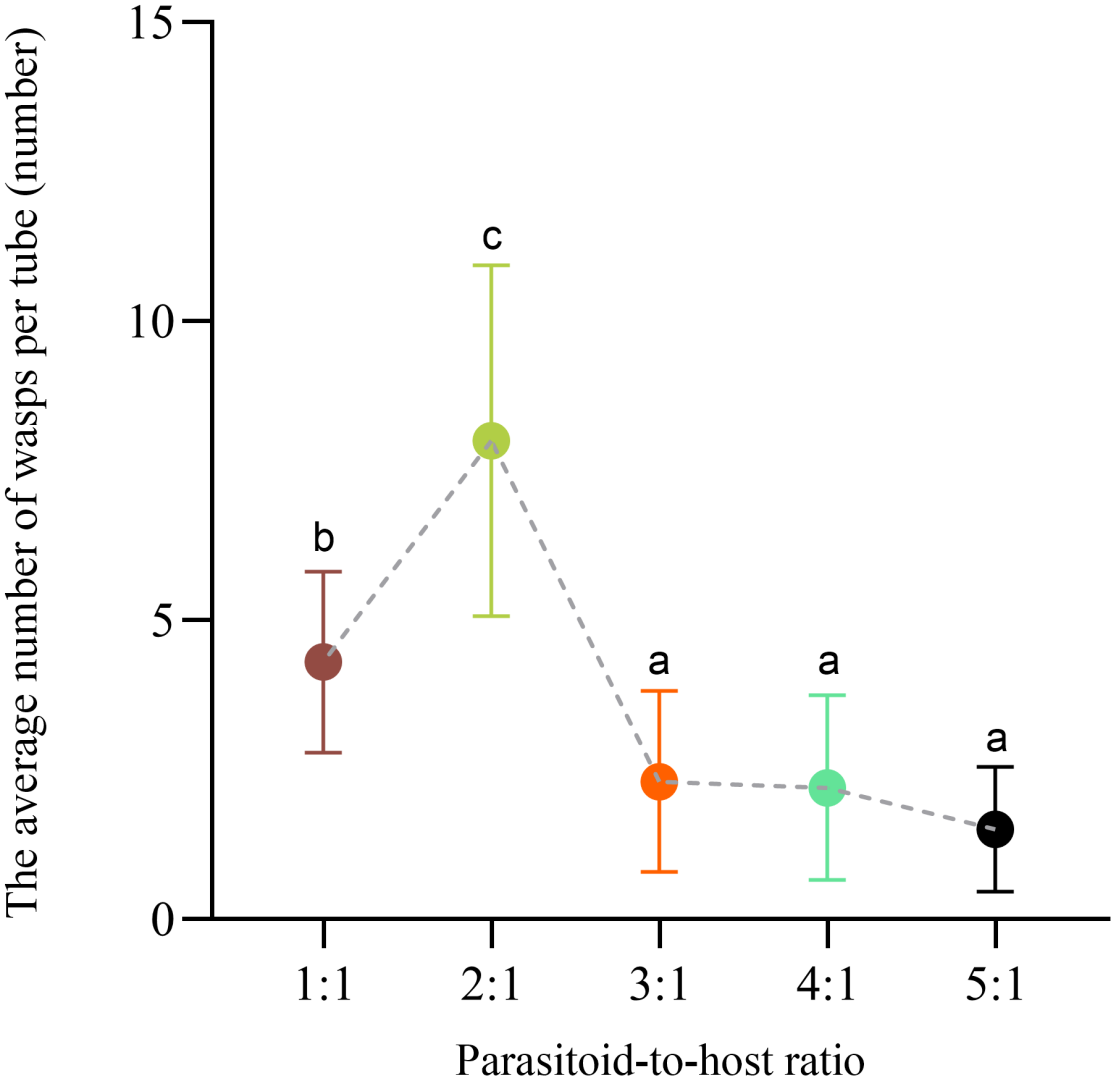
Wasp emergence rate and the average number of wasps per tube under different parasitoid‐to‐host ratio. Each treatment group contained 30 biological replicates; significant differences among different parasitoid‐to‐host ratios are indicated by different superscript letters. Data represent mean ± standard error (*p* < 0.05, Tukey's HSD test).

In this study, the emergence rate and wasp output of *S. sichuanensis* were studied at different parasitoid‐to‐host ratios showed that a parasitoid‐to‐host ratio of 2:1 was the optimal choice (the wasp emergence rate was 73.33% and total wasp output was 240 head, Figure 1, *p* < 0.05). Therefore, in the subsequent experiment, the ratio of bethylid wasps and pupae used was 2:1, that is, each mealworm pupa is parasitized by two wasps.

### 3.2 Part 2: Experiment 2: *T. molitor* Pupae Was Different Ways Anesthesia Under Laboratory Conditions

**FIGURE 2:**
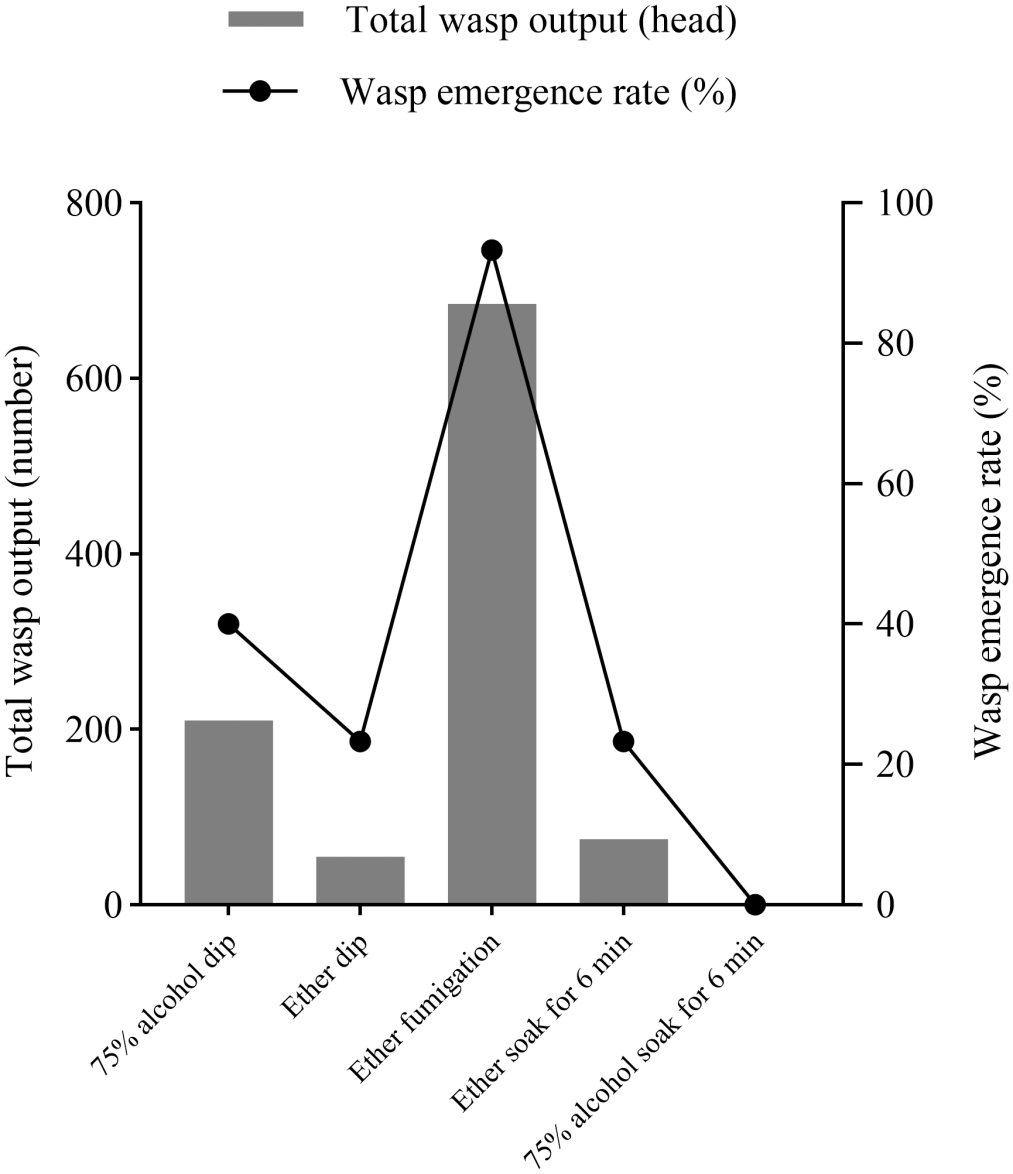
Total wasp output and wasp emergence rate effect of *Tenebrio molitor* pupae treated with different anesthesia methods.

There were differences in the color change in *T. molitor* pupae and the effect on the wasp output of *S. sichuanensis* pupae when different anesthetic methods were used to treat the *T. molitor* pupae (Table 1). As shown in Figure 2, the max wasp output rate (93.33%) and max total wasp output (685 head) of *S. sichuanensis* pupae with feeding *T. molitor* treated with ether fumigation were better than those treated with alcohol, ether dip and soak (Figure 2, *p* < 0.05). When the *T. molitor* pupae were treated with 75% alcohol dip for 6 min, the wasps could not be parasitized. Meanwhile, the body color of the *T. molitor* remained white, and the highest wasp emergence rate was 40.00%; the maximum wasp output from a single tube was 43 head, and the total wasp output was 210 head. Therefore, this treatment was not recommended for the *T. molitor* pupae.

**TABLE 1:** Color change and wasp output effect of *Tenebrio molitor* pupae treated with different anesthesia methods.

Processing	Number of inoculated tubes	Color change of pupae	Is the wasp parasitic	Maximum wasp output from a single tube
75% Alcohol dip	30	White	Parasitic	43
Ether dip	30	White	Parasitic	33
Ether fumigation	30	Brown	Parasitic	41
Ether soak for 6 min	30	Black	Partially parasitic	19
75% Alcohol soak for 6 min	30	Black	Non‐parasitic	0

*Note:* Each treatment group contained 30 biological replicates.

### 3.3 Part 3: Experiment 3: *T. molitor* Pupae Was Duration of Cryogenic Treatment

**FIGURE 3:**
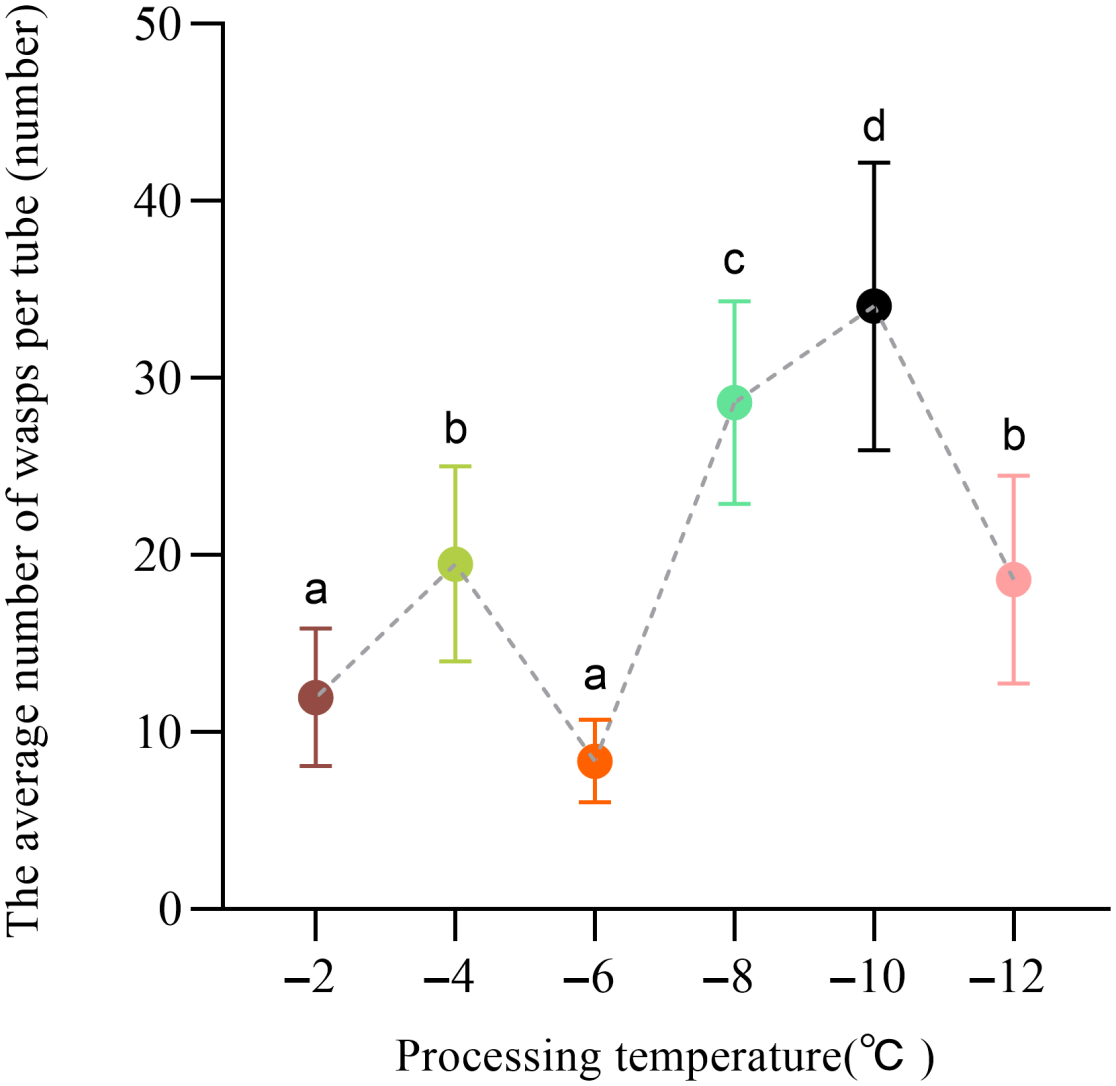
The wasp emergence rate and output of *Scleroderma sichuanensis* after different temperature treatments of *Tenebrio molitor* Each treatment group contained 30 biological replicates; significant difference between the average number of wasps per tube and total wasp output are indicated by different superscript letters. Data represent mean ± standard error (*p* < 0.05, Tukey's HSD test).

After being stored at different temperatures and for different periods of time, the mealworm has lost some of its vitality, which is more conducive to parasitism by the wasp. The wasp emergence rate and wasp output of *S. sichuanensis* under different low‐temperature storage and treatment times are shown in Table 2. The results of bivariate ANOVA showed that there was no difference in the wasp output of *S. sichuanensis* between different treatment times of *T. molitor* pupae (Table 2, *p* > 0.05). However, there is a significant difference in the wasp output *S. sichuanensis* between different temperatures (Figure 3, *p* < 0.05).

**TABLE 2:** The wasp emergence rate and output of *Scleroderma sichuanensis* treated with different temperatures and duration for *Tenebrio molitor*.

Duration (days)	Temperature (°C)					
	0		−4		−8	
	Wasp emergence rate (%)	Total wasp output	Wasp emergence rate (%)	Total wasp output	Wasp emergence rate (%)	Total wasp output
1	26.67	76	73.33	405	93.33	754
2	26.67	179	76.67	585	93.33	858
3	43.33	134	83.33	673	36.67	394

*Note:* Each treatment group contained 30 biological replicates.

As the treatment temperature had a significant effect on wasp emergence, we investigated different temperatures (−2°C, −4°C, −6°C, −8°C, −10°C, and −12°C) to treat *T. molitor* pupae. The parasitoid‐to‐host ratio was 2:1, and the treatment time was 2 days for all treatments to observe the emergence of *S. sichuanensis* on *T. molitor* pupae. As shown in Figure 3, the highest emergence rate (93.33%) and the highest total wasp output (1022 head) were recorded at −8°C and −10°C, respectively. Considering the emergence rate and wasp output together, it was concluded that the treatment temperature of −10°C gave the best results because the emergence rate was 3% lower than that at −8°C, but the total wasp output was 164 head more than at −8°C.

## 4 Discussion

The parasitic wasps of Scleroderma are widely distributed in the world, and the use of parasitic wasps for biological control is a potentially valuable option to control woodborer pests, because woodborer pests are usually hidden in the immature stage of wood, and parasitic wasps have developed morphological and behavioral adaptation to cope with concealed hosts (Quicke et al. 1998; Kenis et al. 2017). In general, biological control plans for invasive alien pests should focus on utilizing the coevolutionary natural enemies of the countries of origin of pests (Kenis et al. 2017). Therefore, we use *T. molitor* as the host to breed the local natural enemy of borer pests, *S. sichuanensis* .

In this research, we discovered that the feed formula is crucial for the growth and development of *T. molitor* . The growth cycle of *T. molitor* is accelerated when balanced nutrition consisting of wheat bran, maize meal, and green fodder feed is employed. (Naser El Deen et al. 2022) results indicate that particle size can significantly influence larval growth and that particles smaller than 2 mm improve larval growth on all feeds except alfalfa pellets. The maximum larval weight was slightly different for wheat bran (12%) when comparing particle sizes smaller than 2 mm with greater than 2 mm but increased up to 70% when corn kernels were used as feed. This also shows that both the type (wheat bran, chicken feed and corn kernels) and particle size (< 2 mm) of the feed were important determinants of larval growth (Naser El Deen et al. 2022). Rizou et al. found that if probiotics were added to the diet of *T. molitor* pupae, there would be a significant improvement in growth enhancement, nutrient fortification and microbial load reduction (Rizou et al. 2022).

Yang et al. found higher offspring wasp emergence rates at 2:1, 3:2, and 4:2 parasitoid‐to‐host ratios, reaching 52.17%, 47.37%, and 47.06%, respectively. There was a higher offspring wasp output at 3:2 and 4:2 parasitoid‐to‐host ratios, with an average output of 44.56 and 45.63 head wasps per tube, respectively, suggesting that the 2:1 parasitoid‐to‐host ratios were the best (Wang et al. 2020; Yang et al. 2017). Therefore, in later experiments, the parasitoid‐to‐host ratio used was 2:1, that is, two *S* *sichuanensis* pupae per *T. molitor* pupa. Wang et al. showed that the parasitism efficiency of each wasp is negatively correlated with the parasitism density. Due to exploitative competition and other reasons, with the increase of the host density, the shared and limited host resources are exhausted. This can also explain why the average number of wasps per tube and wasp emergence rate will decline when more than two wasps parasitize the same *T. molitor* host.

In genetic experiments, ether is widely used to anesthetize *Drosophila melanogaster* , which has the characteristics of fast anesthesia and fast recovery (MacMillan et al. 2017). If the period of anesthesia is too long or the amount of ether used is too much, the pupae of *T. molitor* fumigated with ether will also cause coma of *S. sichuanensis* and damage its parasitism. For the 75% alcohol dip, ether dip and ether fumigation, wasps were parasitized after the treatment of *S. sichuanensis* pupae, and the color change of *T. molitor* pupae were normal. For ether dip and 75% alcohol dip for 6 min, the treated *T. molitor* pupae turned black, and *S. sichuanensis* pupae were partially parasitized, with a strong anesthetic effect on *S. sichuanensis* pupae, which was not conducive to the experiment. Moreover, when the fumigation time of ether was less than 1.5 h, it could not play an anesthetic role in *T. molitor* pupae and did not reduce the activity of *T. molitor* pupae. When the soaking time with 75% alcohol was more than 1 s, the pupae of *T. molitor* turned black, which led to the nonparasitism of the wasp and the wasp being anesthetized to death, which was not conducive to the experiment. The effect of fumigating the pupae with ether to reproduce bethylid wasps was significantly improved, so it is worth promoting the application.

The longer the storage time of mealworms below 0°C, the higher the emergence efficiency of wasps. Low‐temperature treatment will prolong the dormancy time of the *T. molitor* pupae, and help the bethylid wasps bite and sting the *T. molitor* pupae, so as to anesthetize the host, and then lay eggs to complete parasitism. At lower temperatures, it is more helpful to avoid the loss of water and nutrition in the host, and greatly improve the parasitic efficiency of parasitic wasps on the host. If the storage temperature of the host is too high, the host will constantly twist and turn, causing mechanical damage to the bethylid wasps (Tiago et al. 2019).

Although the bethylid wasp has achieved great success in forest control, its control effect can be unstable due to a number of factors, including climatic factors (Jucker et al. 2020). The effect of biological control is slow and cannot be compared with chemical control, and the wasps are parasitized by females, who are basically wingless and search for hosts that are much slower than some winged parasitic wasps. Although the effect of biological control has some shortcomings above that, ecologically speaking, it will not cause environmental pollution and can control pest outbreaks, solving the present pest problems in forests and orchards and thus controlling the ecological balance (Jucker et al. 2020; Wang et al. 2010).

In the study of the artificial reproduction of bethylid wasps, different treatments of the host pupae play an important role in reproduction. The quality of bethylid wasps decreases over many generations, and their ability to survive in the field decreases; therefore, high population fecundity not only improves the quality of bethylid wasps but also the control efficiency. The results of the study showed the wasp emergence rate and production of *T. molitor* pupae at different parasitoid‐to‐host ratios, showing that the 2:1 parasitoid‐to‐host ratio was optimum. When different anesthesia methods were used to treat *T. molitor* pupae, there were differences in the color change of pupae and the laying effect on *S. sichuanensis* . With 75% alcohol dip and ether dip, the color of *T. molitor* pupae remained white, and the Maximum wasp output from a single tube of *S. sichuanensis* was as high as 39%, with the highest wasp output of 43 from a single tube and the total laying capacity of 210. However, if the soaking time of 75% alcohol was longer than 6 min, *T. molitor* pupae would be black, resulting in no parasitism of the majority of wasps and a small proportion of the wasps were fumigated to death, because of the strong odors. The efficacy of the percentage parasitism and the number of outputs of *S. sichuanensis* is closely related to the diet in the prelaying period. Significant differences were found between different low‐temperature treatments, with the highest total wasp output emergence at −10°C in each wasp per tube. Low‐temperature treatment prolongs the dormancy period of the pupae, successfully improving the percentage parasitism, which easily bites and stings the pupae to anesthetize the host and complete the parasitism cycle (Beckage 1985). Lower temperatures are more conducive to preventing pupal water and nutrient loss and greatly increase the parasitism efficiency of the parasitoid wasp on the host. If the host is stored at an elevated temperature, it will exhibit a tendency to twist and turn, which will inevitably result in mechanical damage to the wasp (Lauziere, Pérez‐Lachaud, and Brodeur 2000). Collectively, this information leads us to conclude that although intrinsic factors of *S. sichuanensis* that can change the variation of the ratio in the percentage parasitism and number of outputs, the process of treating host pupae is highly efficient, resulting in the production of artificially reproduced *S. sichuanensis* that serve as a valuable reference in the field of biological control.

## Author Contributions

**Jiangwei Zheng:** visualization (equal), writing – original draft (equal). **Ke Li:** data curation (equal). **Wei Li:** writing – review and editing (equal). **Wensheng Xia:** conceptualization (equal). **Yao Zhao:** supervision (equal). **Tao Liu:** writing – review and editing (equal). **Yu Peng:** project administration (lead), writing – review and editing (lead).

## Conflicts of Interest

The authors declare no conflicts of interest.

## Supporting information


**Table S1.** The average number of wasps per tube at different parasitoid‐to‐host ratios and host pupae cryogenic.

## Data Availability

All study‐related data are available in Table S1.
